# Effect of Elderberry Extract as a Functional Filler on the Physicochemical Properties and Storage Stability of HHP-Induced Pea Protein–Psyllium Hydrogels

**DOI:** 10.3390/gels12100942

**Published:** 2026-10-21

**Authors:** Anna Florowska, Adonis Hilal, Michał Świeca, Małgorzata Sierocka, Tomasz Florowski, Patrycja Wardaszka, Małgorzata Wroniak

**Affiliations:** 1 Department of Food Technology and Assessment, Warsaw University of Life Sciences–SGGW, 166 Nowoursynowska Street, 02-787 Warsaw, Poland; adonis.hilal@gmail.com (A.H.); malgorzata_wroniak@sggw.edu.pl (M.W.); 2 Laboratory of Enzymology and Bioactive Additives, Department of Food Chemistry and Biochemistry, University of Life Sciences in Lublin, 8 Skromna Street, 20-704 Lublin, Poland; michal.swieca@up.edu.pl (M.Ś.); malgorzata.sierocka@up.edu.pl (M.S.)

**Keywords:** hydrogels, pea protein, psyllium, functional filler

## Abstract

The aim of this study was to investigate the effect of elderberry extract on the physicochemical properties and storage stability of HHP-induced pea protein–psyllium hydrogels. Hydrogels were prepared with the addition of 2 g of dry elderberry extract per 100 mL of water used for hydrogel preparation (E2) and without its addition (E0). Hydrogels were tested after 1 and 30 days of refrigerated storage (4 °C). It was found that the addition of the extract did not affect the ability of the pea protein–psyllium system to form a hydrogel network. However, its stability increased after storage (VGI: E0 88%; VGI E2 90%). Hydrogels with the extract added were also characterized by lower fluidity (solid–liquid balance (SLB): E0 0.44; E2 0.37) and higher elasticity (elasticity index (EI): E0 0.022 nm^−2^; E2 0.030 nm^−2^) and macroscopic viscosity (MVI: E0 0.029 nm^−2^ s; E2 0.044 nm^−2^ s), as well as higher adhesion force (E0 0.04 N; E2 0.09 N) and lower spreadability (E0 2.3 N s; E2 3.5 N). It was also observed that the rheological properties of hydrogels with the addition of the extract strengthened after 30 days of storage. Among the microrheological parameters, a decrease was observed in SLB and FI (fluidity index), along with an increase in EI and MVI. Among the texture parameters, an increase was seen in strength, viscosity index, and adhesiveness force. The addition of the extract also affected the color of the hydrogels, causing a decrease in brightness (L*: E0 71.64; E2 6.60), an increase in the share of red color (a*: E0 4.48; E2 12.67), and a decrease in the share of yellow color (b*: E0 16.63; E2 0.41). At the same time, elderberry extract dyes immobilized in the pea protein–psyllium hydrogels underwent relatively few changes during storage (ΔE = 0.53). An important nutritional effect of introducing elderberry extract into hydrogels was the improvement of their phenolic and antioxidant profiles. The addition of this functional filler to pea protein–psyllium hydrogels allowed the maintenance of total anthocyanins during cold storage. Such hydrogels can be used to create new types of food with increased health value and stability.

## 1. Introduction

Innovative food matrices that integrate desirable sensory attributes with health-promoting bioactive compounds are gaining increasing attention in response to the growing consumer demand for functional food products [1,2,3]. Among these bioactives, polyphenolic extracts derived from berries have attracted significant interest due to their well-established antioxidant and anti-inflammatory properties [4,5,6,7,8]. Elderberry (*Sambucus nigra* L.) extract is characterized by a high anthocyanin content, especially cyanidin-3-glucoside and cyanidin-3-sambubioside [9,10]. These anthocyanins are responsible for the fruit’s characteristic deep purple color and are associated with potential protective effects against chronic diseases, mainly through their ability to mitigate oxidative stress [11,12,13,14]. However, incorporating elderberry anthocyanins into functional food systems presents notable formulation challenges, as these compounds are susceptible to degradation under typical processing and storage conditions, including elevated temperatures, pH fluctuations, and prolonged exposure to light or oxygen [15,16,17]. Degradation can result in color deterioration and functional losses, such as reduced antioxidant capacity, diminishing the health benefits of the final product. Therefore, there is a critical need for formulation strategies that can stabilize anthocyanins and preserve their bioactivity throughout a product’s shelf life. In recent years, delivery systems based on proteins and polysaccharides have emerged as promising candidates for this purpose. As a result, developing such matrices has become a key focus in advancing the next generation of elderberry-enriched functional foods [18,19,20].

Among various delivery systems explored for this purpose, particular attention has been given to food-grade hydrogels, which have emerged as promising systems for entrapping and protecting bioactive compounds due to their excellent water-holding capacity and ability to form stable three-dimensional networks [21,22]. These gels are typically formed through the cross-linking of proteins, polysaccharides, or their combinations, allowing them to effectively trap water-soluble nutrients or phytochemicals. This structural capacity not only enhances the stability of sensitive compounds but also helps minimize degradation under complex food processing and storage conditions. Beyond their entrapment and protective functions, hydrogels also play a crucial role in modifying the texture and mouthfeel of food products, making them valuable in the development of functional foods [23,24]. While numerous protein–polysaccharide hydrogel systems have been explored for improving physicochemical properties, the combination of pea protein with psyllium husk—a mucilaginous fiber with high viscosity and water-binding capacity—has received relatively little attention. Most of the existing studies examine pea protein and psyllium husk individually [25,26], rather than exploring their synergistic behavior in a binary hydrogel matrix. One of the few studies addressing the combined use of these biopolymers was conducted by Chen et al. [27]. The authors in their studies on the gelation mechanism of pea protein and psyllium husk stated that the gelation properties of the system containing both components were improved due to several molecular interactions between these biopolymers, including hydrophobic interactions, hydrogen bonding, and electrostatic repulsion. Furthermore, our previous studies [28,29,30] demonstrated that sequential gelation induced by thermal treatment followed by high hydrostatic pressure (HHP) can further enhance the physicochemical and structural properties of pea protein–psyllium hydrogels, indicating that assisted induction is an effective strategy for tailoring the characteristics of these binary systems. What remains underexplored is whether incorporating elderberry extract into a pea protein–psyllium hydrogel network will enhance the system’s physicochemical properties or potentially disrupt its structure by interfering with protein–polysaccharide interactions. This question is especially relevant for matrices designed to entrap and protect anthocyanins during processing and storage, as achieving both functional delivery and desirable texture depends on the behavior of the entrapped extract (filler) within the hydrogel matrix. Although oil droplets are the most extensively studied dispersed phase in emulsion-based hydrogels [31,32,33], hydrogel matrices can also incorporate other types of dispersed materials. These include solid fillers, such as starch granules [34], cellulose [35], and agro-industrial residues [36], as well as aqueous plant extracts containing bioactive compounds [37]. However, the latter remain comparatively underexplored. These fillers can be broadly categorized as passive or active, depending on the nature and strength of their interactions with the hydrogel matrix. Passive (or unbound) fillers do not establish pronounced interactions with the polymer network and primarily act as space-occupying elements. Although they rarely improve the mechanical strength of hydrogels, they can modify their physicochemical properties, such as smoothness, viscosity, and spreadability. Depending on their size and distribution, passive fillers may also reduce the elasticity of the gel by disrupting the continuity of the polymer network [38,39,40,41].

In contrast, active (or bound) fillers establish specific interactions with the surrounding hydrogel matrix, including covalent bonds, electrostatic attractions, and hydrogen bonding. These interactions reinforce the polymer network, leading to improved mechanical strength and structural stability, although excessive reinforcement may also increase gel brittleness. Beyond modifying the hydrogel structure, active fillers can also serve as functional components capable of interacting with or releasing bioactive compounds in response to environmental or physiological stimuli. Owing to these properties, hydrogels containing active fillers are widely used in drug delivery systems, tissue engineering, and regenerative medicine [42,43].

In food systems, a particularly relevant group of active fillers comprises plant-derived bioactive compounds, such as anthocyanin-rich extracts. Recent studies have highlighted the importance of stabilizing anthocyanins in food matrices. However, these studies have primarily focused on preserving the stability and bioactivity of anthocyanins, whereas considerably less attention has been paid to how the incorporation of such active fillers influences the formation, structural integrity, and physicochemical properties of the surrounding hydrogel matrix. Notable gaps remain in the available literature, especially regarding research concerning the utilization of binary pea protein–psyllium husk hydrogels as carriers of anthocyanin-rich elderberry extract. Specifically, little is known about how such systems can limit the molecular mobility and oxidative degradation of polyphenols, particularly under storage conditions, while also maintaining their structural integrity. This gap points to the need for further investigation into the potential synergistic interactions between anthocyanin-rich elderberry extract and the pea protein–psyllium network, and how these interactions affect the hydrogel’s physicochemical behavior over time. Therefore, the aim of this study was to investigate how incorporating elderberry extract affects the physicochemical properties of pea protein–psyllium hydrogels, including their structural stability, microrheological behavior, textural characteristics, and color, over 30 days of cold storage. Particular attention was paid to the stability of anthocyanins and the antioxidant potential of the hydrogels in relation to their potential bioaccessibility. By simultaneously evaluating the stability of both the hydrogel matrix and the incorporated bioactive compounds, this study contributes to the development of next-generation functional foods and supports the design of stable, bioactive-rich hydrogel delivery systems.

## 2. Results and Discussion

### 2.1. Physical Stability

It was found that the addition of dry elderberry extract had no significant effect on the formation of the gel network of HHP-induced pea protein–psyllium hydrogels. Its introduction into the system did not have a negative impact on VGI values (Table 1). Both in the case of the control hydrogel (E0T1D) and with the addition of the extract (E2T1D), the values were 99%, indicating the formation of a gel system with a homogeneous structure. Storage of hydrogels for 30 days resulted in a statistically significant reduction in the VGI, which was the result of the syneresis phenomenon in both systems, i.e., those produced without and with the addition of dry elderberry extract. However, the interactions between the addition of the extract and storage had a significant impact on the stability of the tested hydrogels (E·T η^2^ = 0.990). In the systems containing elderberry extract (E2T30D), the decrease in the VGI was significantly lower (approximately 90%) than that observed for the control system (E0T30D, approximately 88%). This suggests that the addition of dry elderberry extract had a positive effect on the stability of the system during storage. The incorporation of elderberry extract into biopolymer-based gel systems has been reported to affect their structural and physicochemical properties, suggesting that interactions between the bioactive compounds and the components of the gel matrix may contribute to changes in gel stability [44]. In particular, phenolic compounds and other bioactive compounds present in elderberry extract can interact with proteins through hydrogen bonding, hydrophobic interactions, and other noncovalent forces, thereby affecting protein aggregation and the organization of the resulting network [45]. Such interactions may promote the formation of a more cohesive gel structure, increasing its ability to retain water and consequently limiting syneresis during storage.

Analyzing the effect of the addition of dry elderberry extract on the ability of hydrogels to retain water, it was found that the introduction of this additive into the system had no significant effect on the WHC index values, both immediately after their preparation (E0T1D: 61.7%; E2T1D: 62.8%) and after 30 days of storage (E0T30D: 66.1%; E2T30D: 68.1%). Only a significant increase in the WHC index was observed after storage of the hydrogels, which may result from a decrease in the system’s water content due to syneresis. Syneresis is a common phenomenon in gel systems and results from the expulsion of water from the three-dimensional polymer network during storage, which may lead to changes in the water distribution within the gel structure [46].

### 2.2. Microrheological Properties

**Figure 1:**
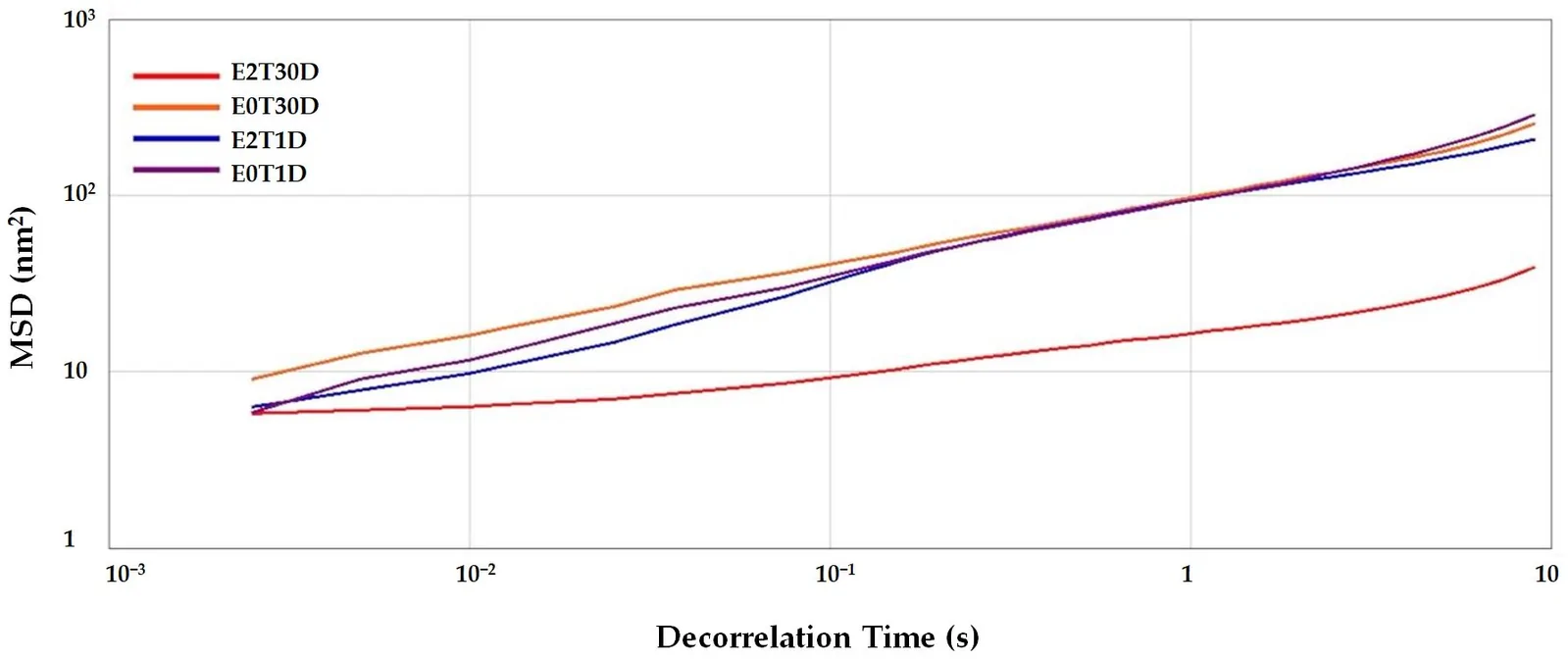
Mean square displacement (MSD) curves as a function of the decorrelation time of the HHP-induced pea protein–psyllium hydrogels.

The microrheological properties of the HHP-induced pea protein–psyllium hydrogels were determined based on the analysis of mean square displacement (MSD) curves as a function of decorrelation time (Figure 1). When comparing the E0T1D and E2T1D curves, the addition of dry elderberry extract does not reduce the mobility of gel particles. Over storage, the presence of extract leads to a large MSD suppression (E2T1D and E2T30D), indicating network aging toward a more solid-like state driven by extract–matrix interactions. A lower MSD indicates a higher viscoelastic modulus for E2T30D, indicating a stiffer, more elastic, and denser hydrogel network with 2 g of dry elderberry extract per 100 mL of water used for hydrogel preparation after 30 days of storage. Such changes were not observed in hydrogels produced without the addition of extract. During storage, the control samples show a slight increase in MSD (E0T30D and E0T1D), consistent with softening and structural relaxation. Based on the results, polyphenol–psyllium/pea protein interactions promote a more cohesive, elastic network during storage, whereas controls undergo mild relaxation.

Based on MSD curves as a function of the decorrelation time of the HHP-induced pea protein–psyllium hydrogels, the following microrheological parameters were also determined: the solid–liquid balance (SLB), elasticity index (EI), and macroscopic viscosity index (MVI) (Table 2). It was found that the addition of dry elderberry extract caused a statistically significant reduction in the SLB index of the hydrogels, and these differences were observed both on the first day after their production and after 30 days of storage (Table 2). Lower values of the SLB index indicate an increase in the elasticity of the system and a shift of its properties in the direction typical for solid samples. In the hydrogel with the addition of dry elderberry extract, a statistically significant reduction in the SLB index was also observed during storage, from 0.37 to 0.30. This effect was not observed in the control systems (E0T1D: 0.44; E0T30D: 0.45). Introduction of pea protein–psyllium hydrogels with dry elderberry extracts resulted in a statistically significant increase in the elasticity index (EI) and the macroscopic viscosity index (MVI) of the systems. This indicates that, thanks to the addition of this functional filler, the resulting hydrogels were characterized by greater elasticity and viscosity, probably due to a more cross-linked structure limiting the mobility of particles in the system. These differences were visible both 1 day after the systems were obtained and after 30 days of storage. At the same time, it was found that storing hydrogels for 30 days resulted in statistically significant increases in EI and MVI values, and statistically significant interactions were observed between the addition of dry elderberry extract and storage in determining the microrheological parameters of hydrogels (EI η^2^ = 0.990 and MVI η^2^ = 0.938). In hydrogels with added extract, the increases in the elasticity index and macroscopic viscosity index during storage were significantly greater than in the control system without the addition of the extract. For the EI, the difference between E0T1D and E0T30D was 0.012 nm^−2^, while the difference between E2T1D and E2T30D was 0.048 nm^−2^. In the case of the MVI, the difference between E0T1D and E0T30D was 0.052 nm^−2^·s, while the difference between E2T1D and E2T30D was greater and reached 0.069 nm^−2^·s.

Based on the results, it can be concluded that elderberry extract enhanced the long-term stability of pea protein–psyllium hydrogels, modulating their water retention and rheological behavior, with the most pronounced effects evident after storage. The observed effects of elderberry extract on the physical stability and microrheological behavior of pea protein–psyllium hydrogels can be interpreted in the context of the molecular interactions of polyphenols with hydrogel networks reported in the literature. Polyphenols have been shown to influence hydrogel stability by interacting with polymeric chains, leading to structural rearrangements, shape changes, and even phase transitions within the gel matrix [47]. The extent and nature of these transformations depend on the number of hydroxyl groups in the phenolic molecules: phenols with fewer hydroxyl groups (≤3) tend to diffuse uniformly into the network, promoting bending, whereas highly hydroxylated phenols (>3) interact more strongly, inducing shrinkage without bending [48]. Such interactions may explain the enhanced gel compactness and reduced SLB observed in elderberry-enriched samples, particularly after prolonged storage. At the microrheological level, polyphenols may modify the balance of intermolecular and intramolecular interactions, including hydrogen bonding and hydrophobic interactions, between hydrogel chains and solvent molecules [49]. This modulation contributes to changes in elasticity and viscosity indices, as seen in the increased EI and MVI values of elderberry-containing hydrogels. The variation in polyphenolic composition among different plant sources is also known to generate diverse effects on hydrogel behavior, with compounds such as gallic acid, EGCG, and tannic acid each exhibiting distinct patterns of influence on structural stability and rheological performance [50]. In the case of elderberry extract, which is particularly rich in anthocyanins and highly hydroxylated phenolic compounds, the strong interaction with the hydrogel network appears to reinforce structural stability during storage, while simultaneously promoting higher macroscopic viscosity and elasticity.

**Figure 2:**
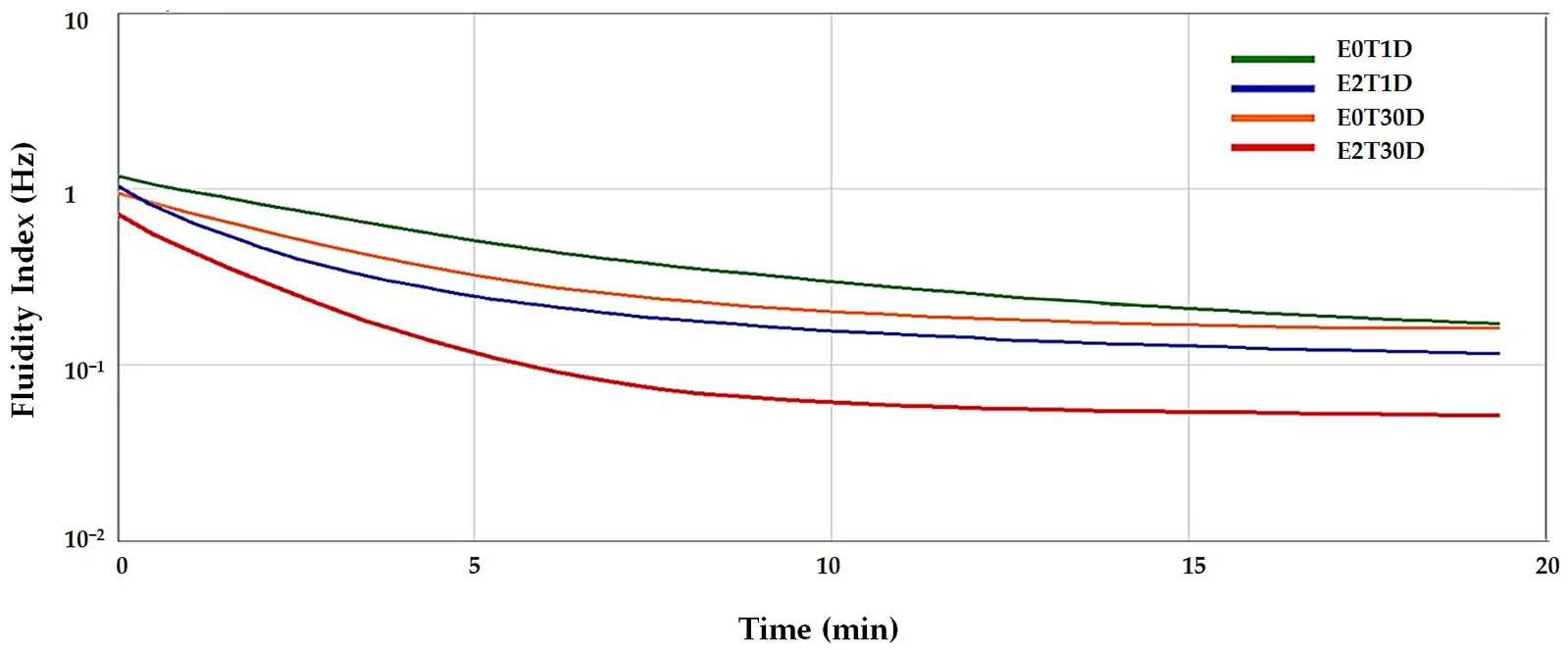
Fluidity index in a function of time in the HHP-induced pea protein–psyllium hydrogels.

The studies conducted also showed the effect of adding dry elderberry extract on the fluidity index of the HHP-induced pea protein–psyllium hydrogels (Figure 2). It was found that the addition of the extract caused a decrease in the fluidity of the hydrogel, as indicated by lower FI values. A lower index indicates stronger elastic dominance and higher structural rigidity. By analyzing the course of the FI curves, it was also observed that, in systems with the addition of dry elderberry extract, 30-day storage resulted in a significant decrease in fluidity (lower E2T30D EI values compared to E2T0D). However, such differences were not observed in the control systems, which indicates the significant role of the extract in changes in the HHP-induced pea protein–psyllium hydrogel structures during storage. The addition of dry elderberry extract influenced the structural properties of the hydrogel network, potentially through polyphenol–polymer interactions. These results are consistent with both the MSD analysis (lower mobility in E2T30D) and textural data (higher viscosity index and strength), reinforcing the conclusion that extract addition combined with storage produces a denser, more elastic, and stable hydrogel network.

### 2.3. Textural Properties

After analyzing the effect of dry elderberry extract on texture parameters of HHP-induced pea protein–psyllium hydrogels, it was found that incorporation of this functional filler into the system had no significant effect on its strength when measured on the first day after preparation (Table 3). However, after 30 days of storage, the hydrogels produced with the addition of the extract were characterized by significantly higher strengths than the control hydrogel. This was the result of a significant increase in the strength of the system after 30 days of storage (from 0.11 N to 0.15 N). A significant increase in strength was also observed in the case of the control hydrogel; however, in this case, the differences were half as large. Therefore, interactions between the presence of the extract in the system and its storage had a significant effect on the strength development of HHP-induced pea protein–psyllium hydrogels (E⋅T η^2^ = 0.915). Increasing the strength of the hydrogel network probably requires a longer interaction time (for the ingredients from the elderberry extract and the protein–polysaccharide network). The addition of the dry elderberry extract also influenced the adhesion strength and spreadability of the hydrogels. The significant differences between the control hydrogel were already observed on the first day after obtaining the gels. Hydrogels with the addition of the extract were characterized by greater adhesion strength (increase from 0.04 to 0.09 N) and lower spreadability (increase in the value of work determined in the spreadability test from 2.3 to 3.5 N·s) than control hydrogels. It was also found that during the storage of hydrogels enriched with the extract, a statistically significant increase in their adhesion strength (an increase of 0.04 N) and a decrease in spreadability (an increase in the value of work of 1.0 N·s) occurred. However, such a statistically significant effect was not observed in the case of the control hydrogel, which indicates that the observed phenomena were the result of the influence of the dry elderberry extract components on the hydrogel network containing pea protein and psyllium. Similar changes during the storage of hydrogels were observed for the viscosity index and extrusion force. The presence of the dry elderberry extract in the protein–polysaccharide matrix resulted in a significant increase in the viscosity index (from 2.58 to 3.59 N·s) and extrusion force (from 170 to 177 N) during the 30 days of storage. Such statistically significant changes were not observed in the control systems. As a result, after storage, hydrogels containing the extract differed significantly from control hydrogels in terms of the discussed texture parameters. It should be noted, however, that on the first day of storage, hydrogels containing dry elderberry extract did not differ significantly from control hydrogels in terms of viscosity index and extrusion force, which indicates that the strengthening of the hydrogel network was necessary not only with the addition of the extract, but also with the time of interaction of the ingredients contained in it with the protein–polysaccharide matrix after 30 days of storage.

The results of the effect of dry elderberry extract on the texture parameters of HHP-induced pea protein–psyllium hydrogels align with previous studies showing that polyphenols significantly influence the mechanical and rheological properties of diverse gel systems through molecular interactions. In several protein-based gels, such as those formed from hazelnut protein isolate or egg white, polyphenols (e.g., EGCG and gallic acid) enhanced gel strength and storage modulus by promoting denser microstructures and stronger hydrogen bonding interactions [47,51]. Likewise, polyphenolic compounds improved the mechanical stability of gellan gum- and gelatin-based hydrogels through hydrogen bonding and hydrophobic interactions [50,52,53,54,55,56,57,58,59]. These mechanisms are consistent with the reinforcement observed in our system, suggesting that polyphenols from elderberry extract may interact both with the psyllium polysaccharide matrix and with pea proteins, thereby stabilizing and consolidating the network.

### 2.4. Color Parameters and Color Difference Coefficient

The primary purpose of designing pea protein–psyllium hydrogels was to improve the stability of the extract during storage by embedding it in a protective hydrogel network. One of the key indicators of physicochemical changes occurring in such systems is color. Based on the research results, it can be stated that the presence of elderberry extract has a dominant effect on hydrogel color (Table 4). ANOVA confirmed that extract addition almost entirely determined color parameters (η^2^ = 0.997–0.999). The addition of dry elderberry extract caused a significant reduction in the color brightness of the hydrogels (reduction in the value of the color parameter L*), an increase in the share of red color (increase in the value of the color parameter a*), and a decrease in the share of yellow color (reduction in the value of the color parameter b*). The color of the hydrogel was a dark, reddish-purple color typical of anthocyanin-rich matrices and differed significantly from the color of the control hydrogel, as indicated by high values of the total color difference parameter (ΔE = 67.73). At the same time, it was found that dyes from elderberry extract set in pea protein–psyllium hydrogels underwent relatively small changes during the 30 days of storage. This is indicated by the low values of the total color difference parameter of hydrogels before and after storage, which are at an average level of 0.53. Such values indicate that the differences in the color of the hydrogels before and after storage were impossible to detect by an observer 0 < ΔE < 1 [60]. After analyzing the values of individual color components of hydrogels with the addition of dry elderberry extract before and after storage, no statistically significant differences were found in the values of the color parameters L* and a*. Significant changes were observed only in the values of the b* color parameter, the share of which decreased slightly. At the same time, these changes could be the result not of changes in the dyes contained in the dry elderberry extract but of changes in the hydrogel matrix itself. In the case of the control hydrogel, a similar, statistically significant reduction in the b* color parameter value was observed during storage. Statistical analysis of the obtained results did not indicate any significant interactions between the tested variable factors, i.e., the addition of extract and storage of the hydrogel, in shaping the color of the hydrogels.

These findings suggest that HHP-induced pea protein–psyllium hydrogels provide an effective protective matrix for elderberry extract, ensuring both color retention and storage stability. This is consistent with previous reports showing that encapsulation of polyphenols within hydrogel networks enhances their resistance to degradation compared with non-encapsulated forms [55,56]. Although limited research has explored pea protein and psyllium husk use in this context, their combined properties appear highly advantageous. Psyllium husk polysaccharides possess unique gelling and self-healing capabilities, enabling the formation of robust networks that immobilize bioactive compounds [57]. Furthermore, the structural integrity of the protein–psyllium matrix and its tunability through pH or ionic strength adjustments [60] contribute to the physicochemical stability of encapsulated polyphenols, including their color characteristics [50,58]. The obtained results confirm that the elderberry extract, when incorporated as a functional filler, not only imparts distinctive optical properties to the hydrogels but also benefits from enhanced stability within the HHP-structured system.

### 2.5. Phenolics and Antioxidant Properties

The incorporation of elderberry extract markedly enhanced the phenolic profile and antioxidant capacity of the pea protein–psyllium hydrogels (Table 5). Hydrogels containing the extract (E2) exhibited a total phenolic content (TPC, CHE) exceeding 5 mg GAE g^−1^, which was more than 37 and 23 times that of the fresh and stored extract-free controls (E0), respectively. Notably, in the tested samples, a slight and insignificant increase in anthocyanin content was observed after storage under refrigerated conditions. These slight differences may be caused by imperfections in the analytical method (pH-differential method) employed; however, it may also be suggested that during storage, the structure of the sample (e.g., the gel, protein matrix, or cellular structure) may have loosened, releasing anthocyanins that were previously undetectable. Similar results were obtained by Plumb [59], who also observed increased anthocyanin content after cold storage of anthocyanin-rich berries. Furthermore, anthocyanins can sometimes migrate from the bound (hydrogel) phase to the liquid phase. Such behavior has previously been reported for kudzu starch hydrogels enriched with rutin and ferulic acid [61]. A definitive explanation for the observed numerical difference would require further investigation beyond the scope of the present study. The enrichment was translated into substantially greater ABTS radical-scavenging activity (≈19 mg TE g^−1^) and reducing power RP (≈8–11 mg TE g^−1^), and confirms the potent antioxidant contribution of the elderberry polyphenols. The chemical extracts from the control hydrogels showed very low phenolics (0.14 mg GAE g^−1^ in fresh samples and 0.25 in stored hydrogels), yet their potentially bioaccessible fractions (PBF) reached ≈1.3 mg GAE g^− 1^. This indicates that in vitro digestion liberated phenolic compounds from the matrix, likely from psyllium husk polysaccharides or minor constituents of the pea protein isolate. It was previously reported in the study of wheat bread fortified with micronized oat and Plantago ovata husks, where digestion also effectively released phenolic antioxidants from supplements used [62]. The high bioaccessibility index (BCI ≈ 5–9) from these controls supports the idea that digestion releases otherwise bound phenolics. Similar results were obtained by Lou et al. (2022) [63], who reported that during in vitro digestion (especially in the gastric phase), a significant release of polyphenols from the matrix occurs, whereas during the intestinal phase, many of them undergo degradation. Collins et al. (2024) also observed that simulated digestion can contribute to an increase in the bioaccessibility of certain polyphenols, for example, as a result of biotransformation (deglycosylation) or their release from the matrix [64]. Interestingly, in digests from the pea protein–psyllium hydrogels without the addition of elderberry extract, total phenolic content (TPC) was significantly higher than in extracts obtained via chemical extraction; however, this increase did not correspond to higher antioxidant activity, likely because the released phenolics can interact with digestive enzymes, decreasing the number of free hydroxy groups traditionally responsible for antioxidant (radical-scavenging efficiency) properties [65]. Additionally, polyphenols released from the protein–polysaccharide matrix may not all be reactive toward the synthetic radicals used in ABTS, DPPH, or RP assays, explaining the lack of correlation between TPC and measured antioxidant activity. Elderberry anthocyanins were well preserved during the 30 days of storage. In the chemical extracts, the values remained around 0.5 mg C3GE g^−1^, suggesting that the pH and hydrogel network effectively stabilized these pigments. The in vitro digestion reduced anthocyanin content to 0.30–0.41 mg g^−1^ (BCI ≈ 0.60–0.9), and the loss was slightly greater in stored samples (E2T30D). This decline was reflected in a lower value of the BCI factor for antioxidant activities (ABTS ≈ 0.60; RP BCI ≈ 0.36). This indicates that storage may subtly alter the anthocyanin microenvironment, making them more susceptible to degradation or transformation during gastrointestinal conditions, especially in the intestinal phase. On the other hand, although interactions between anthocyanins and gel matrix components have not been directly confirmed, the modest decrease in anthocyanin bioaccessibility following prolonged storage may suggest that gradual cross-linking stabilizes these phenolics during digestion. Such a protective effect on phenolics and antioxidant properties was previously described for the mixture of casein and blueberry anthocyanins during intestinal digestion. On the other hand, the observed decrease in anthocyanin bioaccessibility may reflect a reduced recovery resulting from changes in gel structure and protein susceptibility to proteolysis, which could alter anthocyanin release as well as increase their retention in the insoluble fraction. Pineda-Vadillo et al. [66], in studies on anthocyanin-enriched foods, showed that some anthocyanins remain in the insoluble fraction after digestion, leading to an underestimation of their total recovery.

The differences in anthocyanin bioavailability between fresh and stored gels are particularly interesting, and the lack of an in-depth investigation into the underlying mechanisms represents a limitation of the present study that will certainly be addressed in future research. Ultimately, despite the reduced accessibility of antioxidants, the resulting adverse effects are offset by substantially higher initial properties. This study demonstrates that elderberry extract functions as a potent functional filler, substantially improving the phenolic and antioxidant profile of HHP-induced pea protein–psyllium hydrogels while maintaining a high retention of total anthocyanin content during cold storage.

## 3. Conclusions

Elderberry extract can be a valuable addition in the creation of hydrogels with functional properties. Its combination with protein–psyllium hydrogels allows for the production of a food matrix with valuable functional properties resulting from improved phenolic and antioxidant profiles, as well as the creation of hydrogels with a reinforced structure thanks to the interaction of the extract components with the protein–polysaccharide matrix. The combination of these three components also positively affects the stability of such functional hydrogels by limiting syneresis, protecting against color changes, and maintaining a high retention of anthocyanins during storage. HHP-induced pea protein–psyllium hydrogels enriched with elderberry extract can be used to create new types of food with increased health value and storage stability.

## 4. Materials and Methods

### 4.1. Materials

The materials used in this study included pea protein (NUTRALYS^®^ F85F; 88% protein and 10% ash), supplied by Roquette Frères (Lestrem, France), and psyllium husk powder (PS; type 10351; 95% purity; particle size of 60 mesh), purchased from C.E. Roeper GmbH (Hamburg, Germany). The dry elderberry fruit extract (EFE) was provided by GreenVit (Zambrów, Poland). According to the supplier’s specifications, the extract contained 29% anthocyanins and 40% polyphenols, with maltodextrin as the carrier; at least 95% of the material passed through an 80-mesh sieve. Citric acid, sodium citrate, and sodium chloride (NaCl) were obtained from Agnex, a food ingredient supplier based in Białystok, Poland.

### 4.2. Hydrogel Preparation

Hydrogels were prepared according to a previously reported procedure [44], with minor modifications. Pea protein was dispersed in distilled water at a concentration of 12.5 g/100 g of hydrogel and hydrated under continuous stirring at 300 rpm for 60 min at 40 °C using a heated magnetic stirrer (model RCT basic, IKA-Werke GmbH & Co. KG, Staufen, Germany). The resulting dispersion was subsequently maintained at 80 °C for 30 min. After cooling to 20 °C, its pH was adjusted to 3 with citric acid and sodium citrate, and NaCl was added to obtain an ionic strength of 0.3 M. Psyllium husk was then incorporated at 0.5 g/100 g of hydrogel. Two formulations were prepared: a control without elderberry extract (E0T1D) and a formulation containing 2 g of dry elderberry extract per 100 mL of water used for hydrogel preparation (E2T1D). The dispersions were stirred at 300 rpm for a further 10 min before being subjected to high hydrostatic pressure (HHP). High hydrostatic pressure (HHP) treatment was performed using a U5000/120 Pascalizer (Unipress, Warsaw, Poland). The hydrogel samples were placed in 50 mL polypropylene bottles and introduced into the pressure chamber, which was filled with tap water serving as the pressure-transmitting medium at 20 °C. The pressure was increased from 0 to 500 MPa over approximately 150 s. Once the target pressure was reached, it was maintained for 10 min, followed by immediate decompression.

Following HHP treatment, the E0T1D and E2T1D samples were placed in 50 mL polypropylene bottles, filled to minimize headspace, and tightly sealed. One set of samples was stored in darkness at 4 ± 1 °C for 24 h before analysis, while a second set was kept under the same conditions for 30 days. The samples evaluated after 30 days were designated E0T30D and E2T30D. Before physicochemical analyses, all samples were equilibrated to 20 ± 1 °C. Each hydrogel formulation was prepared in triplicate (*n* = 3). The sample coding system is described in Table 6.

### 4.3. Volumetric Gelling Index Measurements

The volumetric gelling index (VGI) was determined to assess variations in hydrogel integrity. This parameter captures both the cohesiveness and stability of the gel network. A VGI of 0% denotes no gelation, whereas a VGI of 100% indicates a fully gelled structure. The following equation was employed in the calculation [67]:

(1) $$\mathrm{VGI}=\frac{V_{G}}{V_{T}}\times 100$$

where V_G_ is the volume of the formulated gel, measured in a glass beaker of the same size and capacity for each measurement, and V_T_ is the total volume of the sample. All results represent mean values from triplicate measurements.

### 4.4. Water-Holding Capacity Measurements

The centrifugation method was used to determine the water-holding capacity (WHC) of the obtained hydrogels [68]. A sample of 5 g of each hydrogel was weighed into test tubes and centrifuged for 15 min at 4584× *g* using a centrifuge (MPW Med. Instruments, Warsaw, Poland). After centrifugation, the resulting liquid phase was separated. The gel remaining in the test tube was then weighed again. The WHC was expressed as the percentage ratio of the mass of the hydrogel remaining after centrifugation to its initial mass before centrifugation. All results represent mean values from triplicate measurements.

### 4.5. Microrheological Measurements

The microrheological behavior of the hydrogels was examined with a Rheolaser Master instrument (Formulaction, L’Union, France), which uses multiple-scattering diffusing wave spectroscopy (MS-DWS) at a near-infrared wavelength of 650 nm. Backscattered light interference patterns were recorded by the detector, and the measurements were processed using Rheotest software (version 1.4.0.114.3). The analysis provided mean square displacement (MSD) curves, and the following microrheological parameters were determined: solid–liquid balance (SLB) [−], elasticity index (EI) [nm^−2^], macroscopic viscosity index (MVI) [nm−2⋅s], and fluidity index (FI) [Hz]. The SLB is defined as the ratio of the viscous modulus (G”) to the elastic modulus (G’). The EI is calculated as the inverse of the MSD value at the plateau and serves as an indicator of elasticity. The MVI corresponds to the apparent viscosity at zero shear rate and is derived from the inverse slope of the MSD curve. The FI is obtained as the reciprocal of the characteristic decorrelation time (τ). Results are reported as means of three independent replicates.

### 4.6. Textural Parameter Measurements

A TA.XT Plus texture analyzer (Stable Micro Mixtures, Surrey, UK) fitted with a 5 kg load cell and designated probes was used to evaluate the hydrogels. Hydrogel strength [N] and adhesion [N] were determined using a cylindrical flat probe (P/0.5R; 0.5 cm in diameter), set to penetrate 8 mm into each sample at 1.0 mm/s. Spreadability [N·s] was determined using a TTC spreadability rig at a speed of 3.0 mm/s. The viscosity index [N∙s] was determined using a back extrusion rig (discs and adapter set—A/BE-D45), and the test speed was set at 1.0 mm/s. Hydrogel extrusion force [N] was determined using a forward extrusion rig (HDP/FE-D) equipped with an insert with a 3 mm Ø outlet (forward extrusion piston disc speed was set at 1.0 mm/s). Data were processed with the Exponent software (version 6.1.4.0, Stable Micro Mixtures, Surrey, UK), and all results represent mean values from triplicate measurements.

### 4.7. Color Parameter Measurements and Color Difference Coefficient Calculation

The surface color of the hydrogels was assessed using a CR-5 colorimeter (Konica Minolta, Inc., Tokyo, Japan) equipped with a D65 light source and an 8 mm measuring head hole) Color was expressed in terms of the CIE L* a* b* coordinates, as described previously [60]. L*, a*, and b* were used to calculate the color difference coefficient (ΔE) according to the following equation:

(2) $$∆E=\sqrt{{\left(L_{1}^{*}-L_{2}^{*}\right)}^{2}+{\left(a_{1}^{*}-a_{2}^{*}\right)}^{2}+{\left(b_{1}^{*}-b_{2}^{*}\right)}^{2}}$$

where $L_{1}^{*}$, $a_{1}^{*}$, and $b_{1}^{*}$ refer to the color parameters of hydrogels either without the addition of elderberry extract (E0) or for 0 storage time (T1) (depending on the calculation), and $L_{2}^{*}$, $a_{2}^{*}$, and $b_{2}^{*}$ refer to the color parameters of hydrogels either with the addition of elderberry extract (E2) or for 30 days of storage time (T30) (depending on the calculation). All results are expressed as the mean of three replicates. The perceptibility of a color difference between samples was assessed according to ΔE values: differences are generally imperceptible when 0 < ΔE < 1, detectable by experienced observers at 1 < ΔE < 2, noticeable by inexperienced observers at 2 < ΔE < 3.5, clearly visible at 3.5 < ΔE < 5, and readily distinguishable at ΔE > 5 [60].

### 4.8. Phenolics and Antioxidant Properties

#### 4.8.1. Extraction Procedures

##### Organic Solvent Extraction (CHE)

The extraction of phenolic compounds from hydrogel samples was carried out following a two-step organic solvent protocol adapted from Bochnak and Świeca [69]. Briefly, 1.00 g of the hydrogel was first homogenized using a mechanical homogenizer [POL-EKO H500, Wodzisław Śląski, Poland]. The homogenized material was then extracted with 10 mL of 1% HCl in 50% (*v*/*v*) methanol and incubated at 4 °C for 30 min on a multi-rotator (RS-60, Biosan, Riga, Latvia) operating at 300 rpm. After the first extraction step, the mixture was centrifuged for 15 min at 6000× *g* (Centrifuge MPW-352R, MPW Med. Instruments, Warsaw, Poland). The resulting pellet was re-extracted with 10 mL of 80% (*v*/*v*) acetone. Supernatants from both steps were combined and stored at −20 °C until further analysis.

#### 4.8.2. In Vitro Digestion (PBF)

The simulated gastrointestinal digestion of hydrogel samples was performed according to the standardized static method described by Minekus et al. [70], with minor modifications. In detail, the hydrogels (1 g) was homogenized in 1 mL of simulated salivary fluid [15.1 mmol/L KCl, 3.7 mmol/L KH2PO4, 13.6 mmol/L NaHCO3, 0.15 mmol/L MgCl2 (H2O)6, 0.06 mmol/L (NH4)2CO3, 1.5 mmol/L CaCl2, and α-amylase (75 U/mL)] and shaken for 10 min at 37 °C. Next, the samples were adjusted to pH 3 with 6 M HCl, suspended in 2 mL of simulated gastric fluid [6.9 mmol/L KCl, 0.9 mmol/L KH2PO4, 25 mmol/L NaHCO3, 47.2 mmol/L NaCl, 0.1 mmol/L MgCl2 (H2O)6, 0.5 mol/L (NH4)2CO3 0.15 mmol/L CaCl2, and pepsin (2000 U/mL)] and shaken for 120 min at 37 °C. After simulated gastric digestion, the samples were adjusted to pH 7 with 1 M NaOH and suspended in 4 mL simulated intestinal fluid [6.8 mmol/L KCl, 0.8 mmol/L KH2PO4, 85 mmol/L NaHCO3, 38.4 mmol/L NaCl, 0,33 mmol/L MgCl2 (H2O)6, 0.15 mmol/L CaCl2, and pancreatin (2 000 U/mL)]. Since the samples contained no lipids, bile salts were not added during the intestinal phase of digestion. The prepared samples underwent in vitro intestinal digestion for 120 min. After completion of the intestinal phase, the digesta were centrifuged at 6000× *g* for 20 min at 4 °C (Centrifuge MPW-352R, MPW Med. Instruments, Warsaw, Poland). The supernatant, representing the potentially bioaccessible fraction (PBF), was immediately mixed with an equal volume of ethanol to terminate enzyme activity and stored at –20 °C until further analysis.

#### 4.8.3. Phenolic Compound Analysis

##### Total Phenolics (TPC)

The TPC was determined using the Folin–Ciocalteu spectrophotometric method [71]. Absorbance was measured at 725 nm (BioTek Epoch2 Microplate Spectrophotometer, Agilent, Santa Clara, CA, USA). Results were expressed as milligrams of gallic acid equivalents per gram of hydrogel (mg GAE g^−1^).

##### Total Anthocyanin (ACN)

Monomeric anthocyanins were quantified by the differential pH method as described previously [72]. Results were expressed as milligrams of cyanidin-3-glucoside equivalents per gram of hydrogel (mg C3GE g^−1^).

#### 4.8.4. Antioxidant Capacity

##### ABTS Radical-Scavenging Activity

The ability to quench ABTS radicals was assessed following Re et al. (1999) [73]. The results were expressed as mg Trolox equivalents per gram of hydrogel (mg TeE g^−1^).

##### Reducing Power

Reducing antioxidant power was determined as described by Oyaizu (1986) [74] and expressed as mg Trolox equivalents per gram of hydrogel (mg TeE g^−1^).

##### Bioaccessibility Index (BCI)

The bioaccessibility index (BCI), indicating the bioaccessibility of active compounds, was calculated using the formula:

(3) $$\mathrm{BCI}=\frac{{\mathrm{PBF}}_{R}}{{\mathrm{CHE}}_{R}}$$

where:

*CHE_R_*—activity of chemical extract/phenolic content in chemical extract;
*PBF_R_*—activity of a potentially bioaccessible fraction/phenolic content in a potentially bioaccessible fraction [75].

### 4.9. Statistical Analysis

Experimental data (*n* = 3) were analyzed using Statistica 13.1 (StatSoft, Kraków, Poland). Two-way analysis of variance (ANOVA) was performed to evaluate the effects of elderberry extract addition, storage time, and their interaction on the measured parameters. Differences between means were assessed using Tukey’s post hoc test, with statistical significance set at α = 0.05.

## Figures and Tables

**Table 1:** Physical stability of the HHP-induced pea protein–psyllium hydrogels (*n* = 3).

Samples	VGI [%]	WHC [%]
E0T1D	99.0 ^c^ ± 0.0	61.7 ^a^ ± 1.1
E2T1D	99.0 ^c^ ± 0.0	62.8 ^a^ ± 0.8
E0T30D	88.0 ^a^ ± 0.0	66.1 ^b^ ± 1.2
E2T30D	90.0 ^b^ ± 1.0	68.1 ^b^ ± 1.5
Statistic ANOVA, η^2^ [-]		
E	0.900	ns
T	0.998	0.869
E⋅T	0.900	ns

a, b, c, etc.—mean values in columns differentiated by letters differ significantly (*p* ≤ 0.05). η2—coefficient indicating the extent of the effect of elderberry fruit extract addition (E), storage time (T), and E⋅T. ns—nonsignificant.

**Table 2:** Microrheological properties of the HHP-induced pea protein–psyllium hydrogels (*n* = 3).

Samples	SLB [-]	EI [nm^−2^]	MVI [nm^−2^⋅s]
E0T1D	0.44 ^c^ ± 0.01	0.022 ^a^ ± 0.000	0.029 ^a^ ± 0.001
E2T1D	0.37 ^b^ ± 0.00	0.030 ^b^ ± 0.001	0.044 ^b^ ± 0.001
E0T30D	0.45 ^c^ ± 0.01	0.034 ^c^ ± 0.001	0.081 ^c^ ± 0.001
E2T30D	0.30 ^a^ ± 0.01	0.078 ^d^ ± 0.002	0.113 ^d^ ± 0.002
Statistic ANOVA, η^2^ [-]			
E	0.979	0.997	0.999
T	0.768	0.995	0.991
E⋅T	0.866	0.990	0.938

a, b, c, etc.—mean values in columns differentiated by letters differ significantly (*p* ≤ 0.05). η2—coefficient indicating the extent of the effect of elderberry fruit extract addition (E), storage time (T), and E⋅T. ns—nonsignificant.

**Table 3:** Textural properties of the HHP-induced pea protein–psyllium hydrogels (*n* = 3).

Samples	Textural Properties				
	Strength [N]	Adhesion * [N]	Spreadability [N∙s]	Viscosity Index [N∙s]	Extrusion Force [N]
E0T1D	0.10 ^a^ ± 0.01	0.04 ^a^ ± 0.01	2.3 ^a^ ± 0.2	2.64 ^ab^ ± 0.05	169 ^a^ ± 1
E2T1D	0.11 ^ab^ ± 0.00	0.09 ^b^ ± 0.01	3.5 ^b^ ± 0.4	2.58 ^a^ ± 0.12	170 ^a^ ± 1
E0T30D	0.12 ^b^ ± 0.01	0.05 ^a^ ± 0.01	2.5 ^a^ ± 0.2	2.81 ^b^ ± 0.00	172 ^a^ ± 3
E2T30D	0.15 ^c^ ± 0.00	0.13 ^c^ ± 0.00	4.5 ^c^ ± 0.3	3.29 ^c^ ± 0.02	177 ^b^ ± 1
Statistic ANOVA, η^2^ [-]					
E	0.787	0.913	0.929	0.792	0.612
T	0.926	0.522	0.637	0.943	0.755
E⋅T	0.915	ns	0.439	0.862	ns

a, b, c, etc.—mean values in columns differentiated by letters differ significantly (*p* ≤ 0.05). η2—coefficient indicating the extent of the effect of elderberry fruit extract addition (E), storage time (T), and E⋅T. ns—nonsignificant. * Absolute value |Adhesion|.

**Table 4:** Color parameters and color difference coefficient of the HHP-induced pea protein–psyllium hydrogels (*n* = 3).

Samples	Color Parameters			Color Difference Coefficient #		
	L*	a*	b*	E2T30D	E0T30D	E2T1D
E0T1D	71.64 ^b^ ± 0.03	4.48 ^a^ ± 0.02	16.63 ^d^ ± 0.02	67.94	0.66	67.73
E2T1D	6.60 ^a^ ± 0.06	12.67 ^c^ ± 0.10	−0.41 ^b^ ± 0.03	0.53	67.14	-
E0T30D	71.15 ^b^ ± 0.01	4.78 ^b^ ± 0.01	16.30 ^c^ ± 0.04	67.35	-	
E2T30D	6.53 ^a^ ± 0.39	12.74 ^c^ ± 0.14	−0.93 ^a^ ± 0.16	-		
Statistic ANOVA, η^2^ [-]						
E	0.998	0.997	0.999			
T	0.421	0.625	0.907			
E⋅T	ns	ns	Ns			

a, b, c, etc.—mean values in columns differentiated by letters differ significantly (*p* ≤ 0.05). η2—coefficient indicating the extent of the effect of elderberry fruit extract addition (E), storage time (T), and E⋅T. ns—nonsignificant. # Color differences were interpreted based on ΔE values as follows: ΔE < 1, imperceptible; 1 < ΔE < 2, detectable only by experienced observers; 2 < ΔE < 3.5, noticeable to inexperienced observers; 3.5 < ΔE < 5, clearly visible; and ΔE > 5, readily distinguishable as two different colors.

**Table 5:** Phenolics and antioxidant properties of fresh and stored hydrogels.

Samples		TPC [mg GAE/g]	ANC [mg C3GE/g]	ABTS [mg TE/g]	RP [mg TE/g]
E0T1D	CHE	0.14 ^d^ ± 0.04	0	0.97 ^d^ ± 0.64	0.19 ^d^ ± 0.09
	PBF	1.32 ^c^ ± 0.07	0	0.33 ^e^ ± 0.23	0.04 ^e^ ± 0.07
	BCI	9.43	-	0.34	0.21
E2T1D	CHE	5.19 ^a^ ± 0.30	0.46 ^a^ ± 0.02	19.56 ^a^ ± 1.06	8.56 ^b^ ± 0.21
	PBF	3.84 ^b^ ± 0.21	0.41 ^b^ ± 0.03	13.47 ^b^ ± 0.70	4.22 ^c^ ± 0.13
	BCI	0.74	0.9	0.69	0.49
E0T30D	CHE	0.25 ^d^ ± 0.13	0	2.09 ^c^ ± 0.07	0.14 ^d^ ± 0.02
	PBF	1.33 ^c^ ± 0.08	0	0.41 ^de^ ± 0.2	0.17 ^d^ ± 0.08
	BCI	5.32	-	0.2	1.21
E2T30D	CHE	5.84 ^a^ ± 0.39	0.50 ^a^ ± 0.02	19.87 ^a^ ± 1.68	10.92 ^a^ ± 0.25
	PBF	3.71 ^b^ ± 0.17	0.30 ^c^ ± 0.01	11.93 ^b^ ± 0.63	3.98 ^c^ ± 0.20
	BCI	0.64	0.60	0.60	0.36

TPC—total phenolics; ANC—total anthocyanins; ABTS—ability to quench ABTS radicals; RP—reducing power; BCI—bioaccessibility index; TE—trolox equivalents; GAE—gallic acid equivalents; C3GE—cyanidin-3-glucoside equivalents; CHE—organic solvent extraction; PBF—potentially bioaccessible fraction. Data are the mean (*n* = 9) ± SD. Values in columns with the same letters are not significantly different (*p* ≤ 0.05).

**Table 6:** Explanation of hydrogel coding.

Sample Code	Elderberry Fruit Extract (EFE) Addition [g/100 mL of the Hydrogel’s Water Phase]	Storage Time [Day]
E0T1D	0	1
E2T1D	2	
E0T30D	0	30
E2T30D	2	

## Data Availability

The original contributions presented in this study are included in the article. Further inquiries can be directed to the corresponding authors.
